# Editorial: Microbial symbionts of lower plants

**DOI:** 10.3389/fmicb.2025.1727008

**Published:** 2025-11-19

**Authors:** Sushma Mishra, Amritesh Chandra Shukla, Kelly D. Craven

**Affiliations:** 1 Department of Botany, University of Lucknow, Lucknow, Uttar Pradesh, India; 2 Department of Biochemistry and Molecular Biology, Oklahoma State University, Stillwater, OK, United States

**Keywords:** microbiome, bryophytes, pteridophytes, symbiotic association, lower plants, evolution

The transition of plants from aquatic to terrestrial habitats represents one of the landmark events in the evolution of life on Earth. Mounting evidence suggests that microbial symbionts—particularly fungi and bacteria—played an instrumental role in helping early land plants adapt to and thrive in the challenging terrestrial environment. These symbiotic interactions likely facilitated nutrient acquisition from primitive soils deficient in organic matter. This symbiotic partnership not only improved plant survival but also played a crucial role in transforming once-barren landscapes into the lush, green ecosystems that characterize our planet today.

Lower plants, including algae, bryophytes, and pteridophytes, represent some of the earliest lineages in plant evolution and occupy distinct ecological niches across diverse habitats. Despite their evolutionary importance and remarkable ecological adaptability, our understanding of their microbial associates remains limited compared to that of angiosperms. To address this knowledge gap, the Research Topic “*Microbial Symbionts of Lower Plants*” was conceived to present new insights into the microbial symbioses associated with bryophytes and pteridophytes. This Research Topic aims to integrate perspectives from microbial ecology, plant–microbe interactions, and evolutionary biology. In essence, this Research Topic, comprising four research articles, highlights a relatively underexplored frontier of plant microbiome research that holds significant promise for advancing our understanding of plant–microbe co-evolution and ecosystem functioning.

The first article explores *bryophyte–microorganism associations* (Dangar et al.), emphasizing the remarkable diversity of microbial interactions within bryophytes (the second most abundant group of land plants after angiosperms). It discusses the roles of bryophyte-associated microbes, particularly bacteria, fungi, and cyanobacteria, in promoting plant growth, enhancing stress tolerance, and facilitating nutrient cycling. This comprehensive synthesis not only consolidates existing knowledge but also highlights key gaps in the understanding of bryophyte microbiology. It is worth noting that bryophytes hold a unique position in the plant kingdom—the closest living relatives of early land plants, avascular plants, the only terrestrial lineage with a dominant gametophytic phase, and a group of immense ecological significance—yet, their associated microbiomes remain largely unexplored.

Moving on from bryophytes, the second article describes the *mycorrhizal associations in pteridophytes* (Kumari et al.). Pteridophytes were the first terrestrial lineage to have evolved the vascular system, and the sporophytic phase as the dominant stage of the life cycle. Both these traits were so advantageous that they have been retained even in the present-day angiosperms. Fossil and molecular evidence suggest that early land plants had symbiotic fungi similar to modern *Glomus* (Glomeromycota). Hence, pteridophytes provide a valuable system to investigate early mycorrhizal functions such as nutrient acquisition and stress resilience, and for understanding the origin and diversification of mycorrhizal partnerships in land plants.

Diving deeper into the pteridophyte-microbe association, the next article describes the *endophytic microbial communities in Alsophila spinulosa*, a tree fern (Chen et al.). The authors present a comprehensive overview of the microbial diversity and composition in different parts of *A. spinulosa* through 16S and ITS-based meta-amplicon sequencing. The study highlights how internal microenvironments (such as moisture, nutrients, available niches etc.) may shape microbial assemblages. The results outlined in the study reveal the complex microbial networks that may contribute to the longevity and adaptability of ferns in forest ecosystems.

Using a similar approach of 16S rRNA gene and transcript sequencing, the *rhizosphere microbiomes of field-grown Boechera stricta* (Ceretto and Weinig) was investigated over diel time (pre-dawn vs. early afternoon) in field conditions. Although *B. stricta* is an angiosperm, its inclusion in this Research Topic is justified by its broader relevance to understanding the stability and functional potential of plant–microbe associations. Besides shedding light on the microbial community dynamics, the researchers also investigated the protein synthesis potential (and hence microbial activity) of the rhizospheric microbes vis-à-vis bulk soil communities. The study demonstrated the stability of rhizosphere communities even under daily environmental changes. This highlights the robust functional associations that sustain plant–microbe interactions in natural settings.

Together, these contributions underscore the ecological and evolutionary significance of plant-microbe symbiotic associations, especially in lower plants. By exploring multiple plant groups and symbiotic strategies, this Research Topic provides a foundation for future studies on functional mechanisms and potential applications in ecosystem management and biotechnology. Lower plant–microbe symbioses remain an underexplored frontier, and continued research in this direction holds promise for unraveling fundamental biological processes and informing climate resilience strategies.

